# Interactions between TGF-β1, canonical WNT/β-catenin pathway and PPAR γ in radiation-induced fibrosis

**DOI:** 10.18632/oncotarget.21234

**Published:** 2017-09-23

**Authors:** Alexandre Vallée, Yves Lecarpentier, Rémy Guillevin, Jean-Noël Vallée

**Affiliations:** ^1^ Experimental and Clinical Neurosciences Laboratory, INSERM U1084, University of Poitiers, Poitiers, France; ^2^ Laboratory of Mathematics and Applications (LMA), UMR CNRS 7348, University of Poitiers, Poitiers, France; ^3^ Centre de Recherche Clinique, Grand Hôpital de l’Est Francilien (GHEF), Meaux, France; ^4^ DACTIM, UMR CNRS 7348, University of Poitiers et CHU de Poitiers, Poitiers, France; ^5^ CHU Amiens Picardie, University of Picardie Jules Verne (UPJV), Amiens, France

**Keywords:** TGF-β, canonical WNT/β-catenin pathway, PPAR γ, radiation-induced fibrosis, myofibroblast

## Abstract

Radiation therapy induces DNA damage and inflammation leading to fibrosis. Fibrosis can occur 4 to 12 months after radiation therapy. This process worsens with time and years. Radiation-induced fibrosis is characterized by fibroblasts proliferation, myofibroblast differentiation, and synthesis of collagen, proteoglycans and extracellular matrix. Myofibroblasts are non-muscle cells that can contract and relax. Myofibroblasts evolve towards irreversible retraction during fibrosis process. In this review, we discussed the interplays between transforming growth factor-β1 (TGF-β1), canonical WNT/β-catenin pathway and peroxisome proliferator-activated receptor gamma (PPAR γ) in regulating the molecular mechanisms underlying the radiation-induced fibrosis, and the potential role of PPAR γ agonists. Overexpression of TGF-β and canonical WNT/β-catenin pathway stimulate fibroblasts accumulation and myofibroblast differentiation whereas PPAR γ expression decreases due to the opposite interplay of canonical WNT/β-catenin pathway. Both TGF-β1 and canonical WNT/β-catenin pathway stimulate each other through the Smad pathway and non-Smad pathways such as phosphatidylinositol 3-kinase/serine/threonine kinase (PI3K/Akt) signaling. WNT/β-catenin pathway and PPAR γ interact in an opposite manner. PPAR γ agonists decrease β-catenin levels through activation of inhibitors of the WNT pathway such as Smad7, glycogen synthase kinase-3 (GSK-3 β) and dickkopf-related protein 1 (DKK1). PPAR γ agonists also stimulate phosphatase and tensin homolog (PTEN) expression, which decreases both TGF-β1 and PI3K/Akt pathways. PPAR γ agonists by activating Smad7 decrease Smads pathway and then TGF-β signaling leading to decrease radiation-induced fibrosis. TGF-β1 and canonical WNT/β-catenin pathway promote radiation-induced fibrosis whereas PPAR γ agonists can prevent radiation-induced fibrosis.

## INTRODUCTION

Patients suffering of cancer often receive an external ionizing radiation therapy in association with surgery/chemotherapy or alone. This ionizing radiation can involve damages in tumor cells but also in healthy tissue in the radiation field. Radiation therapy induces several skin changes such as inflammation, edema, dermatitis, ulceration, late radiation-induced fibrosis (RIF), and necrosis [1, 2]. Radiation dose, fraction size and volume treated will vary the late side effects of radiation.

Radiation-induced fibrosis is marked by fibroblasts proliferation, myofibroblast differentiation, and synthesis of collagen, proteoglycans and extracellular matrix. [3, 4]. RIF can occur 4 to 12 months after radiation therapy, and this process is worsening with time and years. Every radiation-exposed part of the body can be affects by RIF, and the type of tissue exposed will influence the clinical presentation. RIF manifestations are multiple, such as skin induration and thickening, muscle shortening and atrophy, limited joint mobility, lymphedema, mucosal fibrosis, ulceration, fistula, hollow organ stenosis, and pain [5] and generally impact the quality of life [6, 7]. Fibrosis present an active retraction of the granulation tissue which is induced by contractile non-muscle cells, named myofibroblasts [8–10]. Fibroblasts and myofibroblasts are key effectors involved in the development of fibrosis due to excessive deposition of collagen and inappropriate extracellular matrix (ECM).

RIF is characterized by a DNA damage [11–14] and an inflammation [15–18]. Prolonged alteration of nuclear factor kappa B (NF-κB) pathway, a major effector of inflammation, leads to increase pro-fibrotic and pro-inflammatory cytokines which participate to the onset and the progression of RIF [6, 19, 20].

DNA damage and inflammation stimulate transforming growth factor β1 (TGF-β1) activity, which induces fibrosis mechanism [21, 22]. Furthermore, progression of radiation doses increases levels of TGF-β [14, 23].

TGF-β1 interacts with the canonical WNT/β-catenin pathway and peroxisome proliferator activated receptor γ (PPAR γ) which act in an opposite manner in several pathologies [24]. TGF-β1 stimulates myofibroblast differentiation by the activation of canonical WNT pathway and the downregulation of PPAR γ expression [25]. In response to TGF-β1, resident fibroblasts transdifferentiate into contractile myofibroblasts which express α -smooth muscle actin (α -SMA) and synthesize extracellular matrix proteins, particularly collagen.

We focus this review on the positive link between TGF-β1 and canonical WNT/β-catenin pathway and the opposite role of PPAR γ.

### Radiation-induced fibrosis (RIF)

Radiation-induced fibrosis (RIF) is a major late effect which contributes to patient morbidity and occur in the skin, and subcutaneous tissue, lungs, gastrointestinal as well as any other organs in the treatment field. The mechanisms linking radiation to tissue sclerosis, fibrosis and atrophy are complex. In both tumors and normal tissues, radiation causes the induction of apoptosis or clonogenic cell death through free radical-mediated DNA damage [26]. In normal tissues, radiation toxicity induces changes in cell functions and causes activation of coagulation system, inflammation, epithelial regeneration, and tissue remodeling, which are precipitated by several signaling such as cytokines, chemokines and growth factors [27].

#### Etiology of RIF

Several factors can increase the risk of RIF. Total dose of radiotherapy and dose per fraction, volume of tissue treated, and time of treatment delivery are considered as primary factors. Increased levels of radiation dose, hypofractionation and increased field size are directly correlated with degree of RIF [28–32]. Patients with certain diseases are more susceptible to develop severe RIF, such as systemic scleroderma, systemic lupus erythematosus (SLE), or Marfan syndrome [33, 34]. Genetics factors have also a role in the predisposition to RIF [35, 36]. Single-nucleotide polymorphisms have been observed in genes encoding proteins such as TGF-β1, and superoxide dismutase 2 (SOD2) [37, 38]. Additional genes like *IL18* (interleukin 18), *MMP12* (matrix metalloproteinase 12), *PER3* (period circadian protein homolog 3 protein), *LTF* (lactoferrin) stimulate the degradation of post-radiation ECM [39]. Several DNA modifications have been associated with RIF, like epigenetic modifications to DNA and histones [40]. Mitochondrial DNA damage enhance the removal of reactive oxygen species (ROS) [41].

#### Clinical presentation of RIF

RIF usually occurs 4 to12 months after radiation therapy and can progress over many years. The type of tissue exposed to irradiation is responsible for the clinical presentation. In general, RIF can manifest as skin induration and thickening, muscle shortening and atrophy, limited joint mobility, lymphedema, mucosal fibrosis, ulceration, fistula, hollow organ stenosis, and pain [5]. Other manifestations more regionally and specific include trismus, xerostomia, decreased vocal quality, osteoradionecrosis, dysphagia, and aspiration in patients with head and neck malignancy [42–47]; cervical plexopathy, brachial plexopathy, interstitial fibrosis, dyspnea, and oxygen requirement in patients with breast or lung malignancy [48, 49]; and urinary urgency, increased urinary frequency, diarrhea, loss of reproductive function, and dyspareunia in patients with abdominopelvic malignancy [50–52]. Currently, there is no uniform consensus to objectively quantify the degree of fibrosis in RIF [53].

#### Pathogenesis of RIF

Three histopathological phases of RIF are described. The prefibrotic phase shows chronic inflammation in which endothelial cells have a major role. The organized fibrosis phase contains a high density of myofibroblasts in an unorganized matrix adjacent to poorly cellularized fibrotic areas of senescent fibrocytes in a dense sclerotic matrix. The third phase named late fibroatrophic phase shows retractile fibrosis and gradual loss of parenchymal cells [54].

RIF is initially characterized by an injury which incites an acute response leading to inflammation, followed by the accumulation of fibroblasts, differentiation into myofibroblasts, and activation of extracellular matrix proteins like collagen [22]. Radiation induces direct DNA damages and the apparition of reactive oxygen species (ROS) [55] resulting in oxidative stress [56]. ROS involves interactions of ionizing radiation with water molecules and then the formation of free radicals such as superoxide, hydrogen peroxide and hydroxyl radical [57]. Hydroxyl radical production is responsible for the major part of damages [58, 59]. ROS generation and free radicals lead to a deterioration of cellular compounds such as DNA, RNA, proteins, lipids and membranes [58–60]. Superoxide dismutase, glutathione peroxidase and catalase control free radical damages [61]. Several studies have shown that a depletion of these enzymes stimulate oxidative stress [62–64]. During RT, injured cells lead to the release of chemoattractant molecules which can stimulate inflammation [55, 65, 66]. Furthermore, release of inflammatory cytokines and chemokines is exacerbated by thrombosis and ischemia [67, 68].

The first inflammatory cells which arrived at injured sites are neutrophils [69]. Neutrophils encounter fibronectin and collagen fragments and then lead to the release of inflammatory cytokines such as tumor necrosis factor alpha (TNF-α), interleukin 1 (IL-1), and interleukin 6 (IL-6) for the initiation of ROS and local inflammation [3, 70–74]. Theses inflammatory cytokines are correlated with high collagen deposition and with the onset of RIF [19, 75–78]. Monocytes and lymphocytes then interact with injured cells and stimulate the differentiation of monocytes into two subset of macrophages (M1 and M2) [79–81]. Subset M2 of macrophages secrete platelet-derived growth factor (PDGF) which stimulate the migration of fibroblast into injured tissue and the promotion of neo-angiogenesis [82]. Subset M2 of macrophages also secrete TGF-β, which is the main effector of Rif [83]. PDGF and TGF-β cascades are increased in lung tissues after RT [84–87].

TGF-β is responsible for the production of fibroblasts from bone marrow progenitors [88, 89] and for the differentiation of fibroblast into myofibroblasts [14]. The differentiation of fibroblasts results in activation of the expression of α-smooth muscle actin (α-SMA) which is responsible for the transformation of proto-myofibroblasts into matured myofibroblasts [90]. Fibrocytes (bone marrow-derived progenitor cells) and epithelial cells during epithelial-mesenchymal transition (EMT) can also be at the origin of myofibroblasts [91]. Activated myofibroblasts, by overexpression of TGF-β, secrete too much collagen, fibronectin and proteoglycans [92]. This phenomenon is responsible for stiffness and thickening of tissues under RT [68, 93]. In addition, TGF-β stimulates inhibitors of matrix metalloproteinases (MMP) and then decreases the activity of MMP-2 and MMP-9, which participates to the aggravation of excessive ECM deposition [94]. TGF-β stimulates ECM production and collagen architecture alterations [95, 96]. Excessive accumulation of collagen reduces the vascularization with time [68], although basic fibroblast growth factor (bFGF) is activated by myofibroblasts and participates for endothelial cell proliferation and neoangiogenesis [97]. Loss of cell functions, tissue atrophy, and then necrosis are induced by damages and gradual ischemia suffered by radiation-induced fibrosis [49, 65, 98–100]. However, the apparition of early fibrosis is not correlated with the development of lasted effects of RIF [101–103].

### TGF-β and radiation therapy (cf. Figure 1)

Transforming growth factor β (TGF-β) is made of three similar proteins: TGF-β1, TGF-β2 and TGF-β3. TGF-β receptor 1 and TGF-β receptor 2 are transmembrane proteins. TGF-β1 binds TGF-βR2 which recruits TGF-βR1. TGF-β1 is secreted and then deposited into ECM and considered as a large latent complex. ECM is a main reservoir of cytokines [104]. TGF-β1 stimulates the Smad pathway and non-Smad pathways such as phosphatidylinositol 3-kinase/serine/threonine kinase (protein kinase B) (PI3K/Akt), Rho, and MAPK. Radiation-induced fibrosis is characterized by an upregulation of TGF-β1 [21, 23, 84, 105, 106].

**Figure 1:**
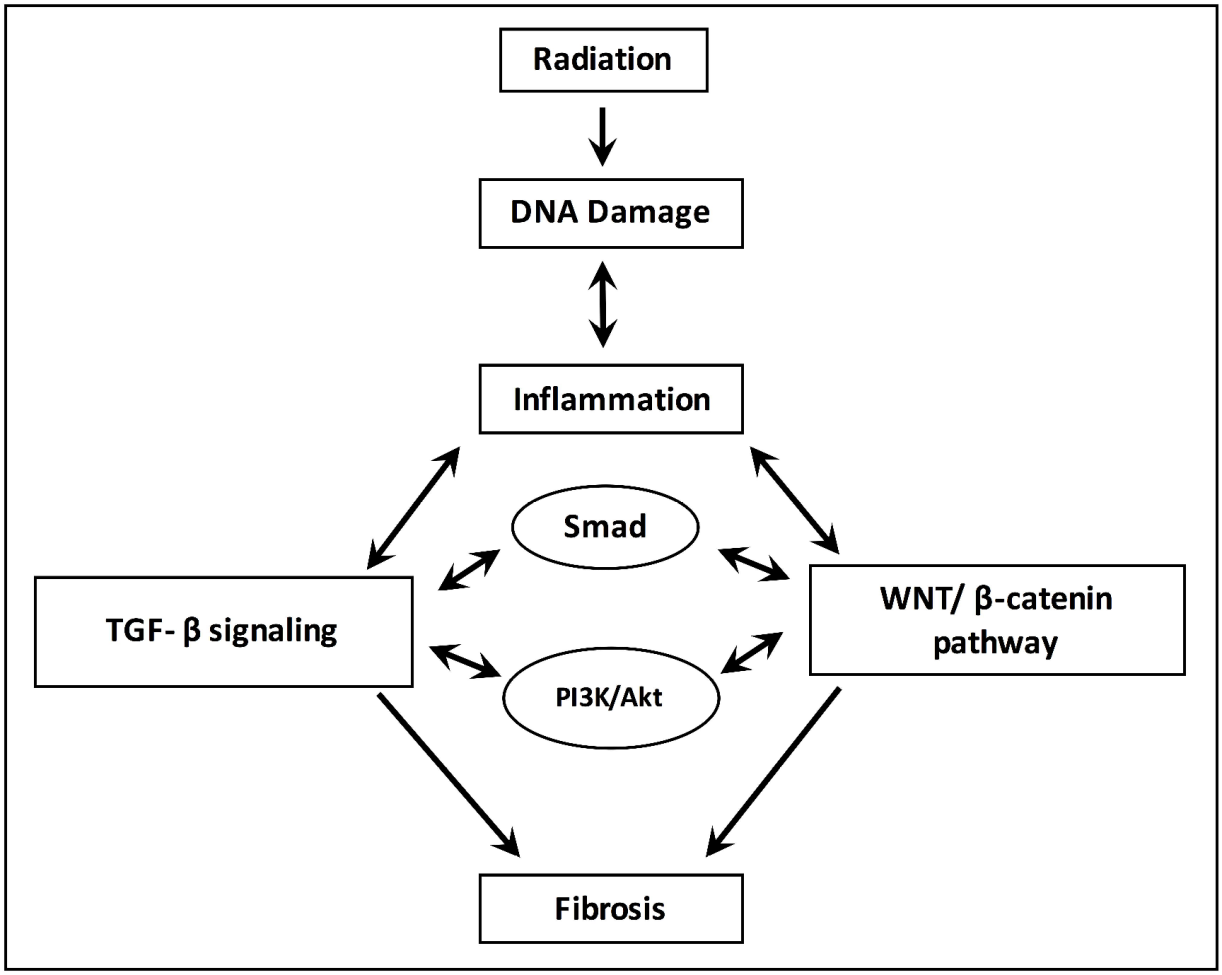
Processes for the development and progression of radiation-induced fibrosis

TGF-β is responsible for the control of many functions, like breakdown of connective tissue, inhibition of epithelial cell proliferation and synthesis of extracellular matrix proteins (collagen and MMPs) [106].

Several studies have shown a weakly activation of TGF-β1 on day 1 after irradiation whereas its activation is strongly on day 14 after irradiation [106]. Thus, the irradiation-induced activation of TGF-β1 is rapid [107–110]. EMT is initiated by a prolonged irradiation exposure to TGF-β1 and this activates several transcriptional signaling pathways [111–113]. Radiation increases fibroblast differentiation-induced alpha-smooth muscle actin (α-SMA) activation [114]. Myofibroblast activation releases α-SMA or myofibroblasts activate the release α-SMA and α-SMA confers to myofibroblasts a strong contractile property [115, 116]. The interplay between TGF-β and several molecules, like extracellular signal-related kinase (EK), mitogen-activated protein kinase (MAPK), WNT, and PI3K, can explain the extent and the reversibility of EMT) [113].

A close relationship is observed between irradiation dose and liver cell viability [117]. Indeed, ionizing irradiation induces a progressive change in mRNA levels which gradually increase with time [118, 119]. Evolution of α-SMA in liver fibrosis induction suggests a time-dependent effect of irradiation [120, 121]. TGF-β1 expression gradually increases in a dose-dependent manner and peaking 4 weeks after irradiation [122].

### The contractile non-muscle cells: myofibroblasts

Myofibroblasts contain actin filaments bundles with α-SMA. α-SMA represents peripheral focal adhesions and gap junctions for the connection between myofibroblats and granulation tissues [123]. Fibroblasts become protomyofibroblats after this interaction.

Protomyofibroblasts synthetize ECM, collagen, fibronectin which are essential for differentiation [124]. Then, protomyofibroblasts differentiate into myofibroblasts containing α-SMA. α-SMA is responsible for the retractile role of myofibroblasts [25]. Apoptosis is the final stage of myofibroblasts [125].

Mesenchymal stem cells (MSC) and fibroblasts are at the precursors of myofibroblasts, and these cells are found in normal tissues, like lung alveolar septa, uterine submucosa, lymph nodes, spleen, adrenal capsule, periodontal ligament, intestinal crypts and bone marrow stroma [115, 125].

Several diseases show myofibroblasts such as idiopathic pulmonary fibrosis and epithelial cancers [116]. In hypertrophic scar, myofibroblasts can persist after wound closure [126]. Precursors of myofibroblasts can be smooth muscle cells in coronary atheromatous plaque [127], perisinusoidal cells in liver [128], keratocytes in eyes [115], pericytes in kidneys [129], and bone marrow-derived fibrocytes [130]. Myofibroblasts are also observed in several pathological tissues like cancers (mammary carcinoma, epithelial cells in cancerous mammary glands), and fibrotic lesions [131].

Moreover, non-fibroblastic cell lineages [132–134] can differentiate into myofibroblasts through EMT process [135]. Differentiation of fibroblasts into myofibroblasts needs the participation of physical and chemical factors with increased stiffness of tissues [90, 136] and association of TGF-β1 with extra domain A (EDA) fibronectin [124, 137]. α-SMA increases contractile property of myofibroblasts and is synthesized through TGF-β1 activation [138]. The transmission of force generated by α-SMA and the molecular motor myosin are allowed by ECM through focal adhesions containing transmembrane integrins [139]. TGF-β1 is activated through an integrin-dependent process in ECM [126].

Myofibroblasts are contractile non-muscle cells, characterized by the type IIA non-muscle myosin (NMIIA) [140]. NMII are responsible for cell polarity, cell migration and cell-cell adhesion. NMIIA are highly observed in myofibroblasts of human placental stem villi [141, 142]. NMIIA is characterized by three pairs of chains, two heavy chains of 230 kDa, two 20 kDa regulatory light chains (RLCs) which stimulate the NMII activity and two 17 kDa essential light chains (ELCs) which stabilize the heavy chain structure. Calcium-calmodulin-myosin light chain kinase (MLCK) and Rho/ROCK/myosin light chain phosphatase increases NMII activity [90, 143, 144].

Myosin filaments link actin filaments in thick bundles like as stress fibers. NMMIIA molecules assemble into bipolar filaments. A title of the head enables a conformational change that moves actin filaments in an anti-parallel manner. The cross-bridge actin-myosin cycle of NMIIA is overall like smooth and striated muscle myosin. An ATP molecule binds the NMIIA-ATPase site on the myosin head. This allows the dissociation of actin from the NMIIA head. ATP is then hydrolyzed and subsequently, NMIIA binds to actin. Then, the power stoke occurs with a tilt of the NMIIA head, which generates a CB single force and a displacement of few nanometers. ADP is then released from the acto-NMIIA complex. A new ATP molecule dissociates actin from myosin head, and a novel CB cycle begins.

However, the major feature of NMIIA is its extreme slowness, with dramatic slow of its kinetics of contractile [145, 146]. The cross-bridge actin-myosin detachment constant, attachment constant, catalytic constant are widely low compared with striated or smooth muscles. Nevertheless, the cross-brigde actin-myosin of NMIIA single force is same order of magnitude compared with muscle myosin II (MII). Thermodynamic force, thermodynamic flow and thermodynamic entropy production rate are rarely low [147]. This explains why this stationary contractile system operates near-equilibrium. The low isometric tension observed in placental stem villi [141, 148] can be surely explained by the low placental myosin content [149, 150]. The extremely slow shortening velocity can be accounted for by the very low placental myosin ATPase activity which has an essential role for the association/dissociation of actin from the NMIIA head [145, 149]. In placental stem villi, a low isometric tension has been reported [147]. This can be explained by the low placental myosin content and the low placental myosin ATPase activity [148]. The acto-myosin apparatus functions as in smooth muscles in myofibroblasts of human placenta. In experimental bath, addition of KCl or electrical field induce the contraction phase. 2,3-butanedione monoxime, or isosorbide dinitrate, two inhibitors of NMII, can induce the relaxation phase [146]. Changes in the volume of the intervillons chamber can alter the length of the placental stem villi. Contraction of myofibroblasts induces a modulation of the distal resistance of the umbilical artery and then a modulation of the umbilical blood flow, due to the Starling phenomenon. In fibrotic processes, myofibroblasts generate rather a phenomenon of contraction-retraction lasting without relaxation and the pathological tissue undergoes an irreversible retraction, evolving towards fibrosis favored by the synthesis of collagen.

### Canonical WNT/β-catenin pathway

Wingless and integration site (named WNT) pathway is a cascade of numerous signaling which are involved in development, metabolism, growth cellular, and maintain of stem cells [151]. WNT pathway is formed by secreted lipid-modified glycoproteins [152]. Deregulation of the canonical WNT pathway is observed in several pathologies [24].

WNT extracellular ligands bind Frizzled (FZD) receptors, low density lipoprotein receptor-related protein 5 and 6 (LRP 5/6) and disheveled (DSH), which lead to stimulate β-catenin accumulation and then the nuclear β-catenin translocation for the bind to T-cell factor/lymphoid enhancer factor (TCF/LEF) [153]. TCF/LEF related to nuclear β-catenin activates numerous target genes such as c-Myc, cyclin D1 [154].

Downregulation of the WNT pathway is characterized by the absence of binding between WNT extracellular ligands and the complex FZD/LRP 5/6. Thus, adenomatous polyposis coli (APC), AXIN and glycogen synthase kinase-3 (GSK-3β) form the β-catenin destruction complex and mediate degradation of β-catenin in the proteasome [155]. GSK-3β inhibits β-catenin accumulation and its nuclear translocation [155, 156].

WNT/β-catenin pathway is increased in fibroblasts in response to radiation during skin fibrogenesis [157]. WNT ligands are activated after irradiation and promote survival in head and neck cancers [158, 159]. Radiation stimulates ERK pathway through a reactive oxygen species (ROS) generation and inactivates GSK-3β [114]. Irradiation inhibits GSK-3β activity in mesenchymal cells [160] and increases WNT genes expression in fibroblasts [161]. Moreover, WNT/β-catenin pathway is overexpressed in liver, skin, lung, kidney, and heart presenting fibrosis [162–167].

Phosphatidylinositol 3-kinase/serine/threonine kinase (protein kinase B)/mammalian target of rapamycin (PI3K/Akt/mTOR) pathway is present in cell growth, proliferation, protein synthesis and energetic metabolism [168–171]. WNT/β-catenin pathway is considered as an upstream activator of PI3K/Akt/mTOR pathway [172] through the inhibition of GSK-3β [173]. In addition, diminution of bet-catenin signaling decreases the expression of PI3K/Akt/mTOR pathway [174, 175]. Moreover, in adipocyte differentiation, activated PI3K/Akt pathway inhibits GSK-3β [176, 177]. WNT/β-catenin pathway and PI3K/Akt/mTOR pathway mediate each other.

### Inflammation and canonical WNT/β-catenin pathway

NF-κB signaling is a main effector of inflammation [178–180], its deregulation is implicated in numerous inflammatory processes [179–181]. Several studies have shown an interplay between canonical WNT/β-catenin pathway and NF-κB signaling [182]. Inflammation and immune response are modulated by this interaction between WNT/β-catenin pathway and NF-κB [182–185].

Overexpression of WNT/β-catenin pathway increases the NF-κB-mediated anti-apoptotic action [186, 187], and activates inflammatory processes through the stimulation of β-catenin targets genes [188, 189]. Activated β-catenin/TCF4 stimulates the NF-κB activity in vascular smooth muscle cells [190] and in colorectal cancer cells [191]. Activated WNT pathway in THP-1 cells induces release of inflammatory cytokines [192]. Nuclear activation of β-catenin associated with p50 and TNF-α, stimulates C-reactive protein (CRP) expression in 293T and HepG2 cells [193]. Nuclear β-catenin coupled with the TCF/LEF complex activates NF-κB target genes, such as CRP and matrix metalloproteinase 13 (MMP-13) in chondrocytes [194, 195], and then induces their genes transcription [193, 194]. In breast cancer, β-catenin/TCF and NF-κB signaling activate each other in a synergistic manner [196]. WNT/β-catenin pathway inhibits prolyl-hydroxylase and then activates NF-κB signaling [197, 198]. GSK3-β upregulation also decreases NF-κB signaling [199, 200].

Moreover, the NF-κB signaling activates canonical WNT/β-catenin pathway [201]. NF-κB activation stimulates the expression of the TCF/LEF complex and provides an indirect positive control of WNT/β-catenin pathway [202]. In human adipose tissue and bone marrow stroma cells, NF-κB decreases the expression of LZTS2 which inhibits β-catenin nuclear translocation and transcriptional activity [203, 204]. Stimulation of IKKB kinase (IKK) α, an activator of NF-κB, leads to cytosolic accumulation of β-catenin and then activates WNT/β-catenin pathway targets genes [201, 205]. Overexpression of interleukin 1 β (IL-1β) in mouse chondrocytes leads to a positive direct binding between NF-κB and LEF to induce β-catenin/LEF transcription [202]. NF-κB-induced TNF-α macrophages stimulates β-catenin cytosolic accumulation [206].

Ionizing radiation activates NF-κB which contributes to cell sensitivity to radiation [207]. NF-κB is activated in endothelial cells after irradiation and is necessary for radiation-induced IL-6 release [208]. Inflammation, through the activation of NF-κB, promotes the production of collagen and the release of inflammatory chemokines in the development of liver fibrosis [209]. NF-κB expression is stimulated by the activation of Akt pathway observed in RIF [210]. In fibrosis, CCN4, a WNT-inducible signaling pathway protein-1 (WISP1) is increased and participates to the fibroblast proliferation and ECM expression [211, 212]. CCN4 overexpression induces morphological transformation in skin fibrosis [213]. CCl4mAb, a specific inhibitor of CCN4, reduces NF-κB activity and then decreases the expression of pro-fibrotic factors, such as TGF-β1, in hepatic fibrosis [214].

### Interactions between TGF-β1 and canonical WNT/β-catenin pathway (cf. Figure 2)

The canonical Smad pathway activates intracellular TGF-β1 signaling in activated myofibroblasts. TGF-β1 binds TGF-βR2 and then interacts with TGF-βR1 to form a heterotetramer for the phosphorylation of Smad2 and Smad3 which binds Smad4. The complex formed translocates to the nucleus for the activation of Smad binding element (SBE) DNA sequences and for recruit coactivators like histone acetyltransferase p300 [215]. Canonical WNT ligands, such as WNT3a, increase TGF-β1 through a Smad2 activation in a β-catenin-dependent manner and lead to myofibroblasts differentiation [216]. A non-Smad pathway is also observed and represented by MAPK, TGF-β activated kinase (TAK1), JNK, or PI3K/Akt [133, 217]. Phosphatase and tensin homolog (PTEN) is an inhibitor of PI3K/Akt pathway [218]. PTEN inhibits myofibroblasts differentiation, and the expression of collagen and α-SMA [219].

**Figure 2:**
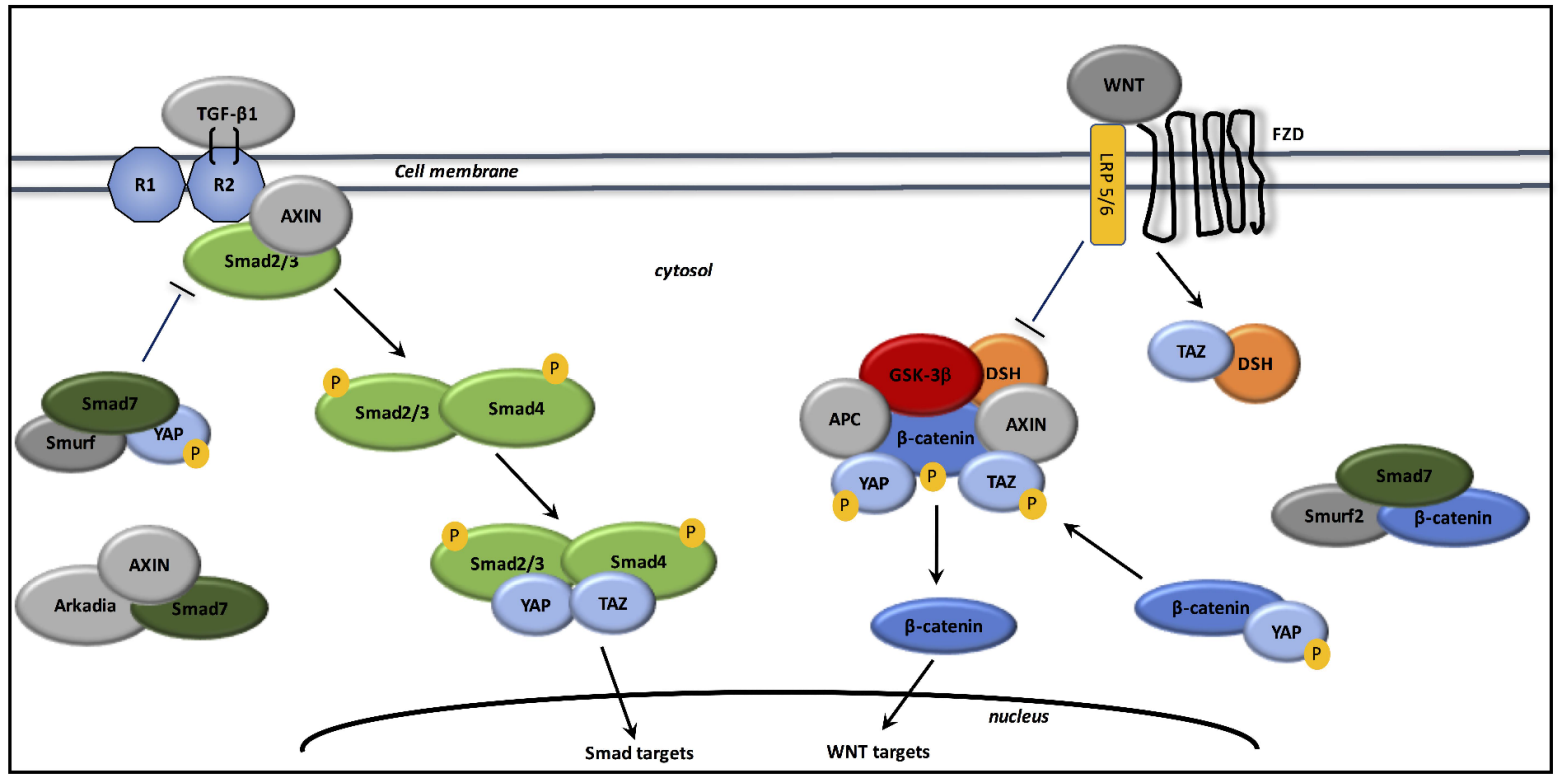
Interactions between TGF-β1, canonical WNT/β-catenin pathway, and YAP/TAZ signaling In the absence of WNT ligands, Smad7 associates with β-catenin and Smurf2 to degrade β-catenin. Smad7 also binds phosphorylated YAP and Smurf to form a high affinity complex for TGF-β type 1 receptor to decrease TGF-β signaling expression. In the absence of WNT ligands, phosphorylated YAP and TAZ are associated with the β-catenin destruction complex. Phosphorylated YAP is also associated with β-catenin to inhibit its nuclear translocation and promote its degradation. Upon WNT stimulation, the beta-catenin destruction complex is inhibited and then beta-catenin accumulates in the cytosol and translocates to the nucleus. TAZ inhibits the phosphorylation of DSH and dissociates it from the β-catenin destruction complex. The destruction complex is inhibited because YAP and TAZ dissociate from the complex. β-catenin accumulates in the cytosol and then translocates to the nucleus for activating WNT targets. Upon TGF-β stimulation, Axin promotes the tail-phosphorylation of Smad2/3. Axin also forms a complex with Arkadia and Smad7 for enhancing TGF-β signaling. The activated Smad complex associates with TAZ and YAP and then translocates to the nucleus for activating Smad targets.

Fibroblasts stimulation with activated WNT3a enhances the expression of TGF-β and then the phosphorylation of the MH2 domain of Smad2 [216]. Conversely, the absence of WNT ligands decreases the expression of TGF-β and then attenuates the fibrotic response [220].

Without tail-phosphorylation by the TGF-β type I receptor, Smad2/3 cannot interact with Smad4 and then cannot engaged the DNA. Smads inactivated are phosphorylated by activated GSK-3β and then degraded [221]. Moreover, activation of TGF-β also enhances WNT pathway stimulation through the inhibition of dickkopf-related protein 1 (DKK1) [222].

Upon TGF-β and WNT stimulation, Axin facilitates the binding of Smad2/3 with TGF-β type 1 receptor and then activates Smad2/3 [223]. Moreover, upon WNT ligands activation, Axin forms a complex with Smad7 and the E3 ubiquitin ligase Arkadia to promote the degradation of Smad7 [224].

Smad7 is an inhibitor of Smad pathway [225]. Activated Smad7 binds YAP (yes-associated protein 1) and Smurf to increase affinity for TGF-β type 1 receptor, and then decreases TGF-β signaling [226]. Activated Smad7 also recruits Smurf 2 to induce ubiquitination and degradation of β-catenin [227].

Myofibroblast activation and fibrosis induction have been recently translated by the mechanical properties of YAP and TAZ. YAP and TAZ (transcriptional coactivator with PDZ-binding motif) elevated levels are observed in fibrosis [228]. YAP and TAZ knockdown in lung and liver fibroblasts cultured reduces the levels of protein associated with myofibroblast differentiation, such as pro-collagen and α-SMA [229]. During fibrosis, F-actin polymerization inactivates the Hippo core kinase complex leading to the dephosphorylation of YAP and TAZ [230].

Stimulation by WNT3a inhibits the destruction complex because YAP/TAZ dissociates from the β-catenin destruction complex and then allowed β-catenin nuclear translocation. TAZ binds DSH and dephosphorylates it upon stimulation with WNT3a to dissociate DSH from the destruction complex [231]. Indeed, in the absence of WNT ligands, phosphorylated YAP and TAZ bind β-catenin with activated GSK-3β and Axin to degraded β-catenin in the proteasome [232]. In fibrosis, activated Smad2/3-Smad4 complex is associated with YAP and TAZ for its translocation to the nucleus [233]. A crosstalk of several components of the TGF-β, WNT, and YAP/TAZ signaling is playing in the tuning of nucleocytoplasmic shuttling of fibrosis [230].

### PPAR γ

Peroxisome proliferator receptor γ (PPAR γ) is a ligand activated transcriptional factor which forms a heterodimer with retinoid X receptor (RXR) to activate specific peroxisome response elements (PPRE) [234]. PPAR γ expression is involved in several mechanisms such as glucose and lipid metabolism, immune response, and inflammation [235, 236]. PPAR γ decreases NF-κB activity and then represses inflammation [237]. PPAR γ is expressed in several cells, such as adipocytes, muscle cells, brain cells, immune cells and in fibroblasts [238].

15d-prostaglandin J2 (15d-PGJ2), lysophosphatidic acid, and nitrolinoleic acid are natural activators of PPAR γ [239], whereas thiazolidinediones (TZDs) and oleanic acid derivatives such as triterpenoids (2-cyano-3,12-dioxoolean-1,9-dien-28-oic-acid (CDDO)) are synthetic activators of PPAR γ. PPAR γ expression mediates the functions of many signaling such as connective tissue regulation, mesenchymal cell activation, differentiation and cell survival creating a link between metabolism and fibrogenesis [134].

Numerous inflammatory cytokines, chemokines, or intracellular signaling decrease PPAR γ expression such as TFG-β1, canonical WNT/β-catenin pathway, TNF-α, interleukin (IL)-1, IL-13, Connective Tissue Growth Factor (CTGF), leptin, and lysophosphatidic acid (LPA) [240–242]. The transcription factor COUP II is a canonical WNT target and represses PPAR γ expression [243]. In adipocytes, adiponectin increases PPAR γ expression and decreases LPS-induced NF-κB expression and IL-6 production [244].

#### PPAR γ and fibrosis

An inverse relationship is observed between the expression of PPAR γ and the apparition of fibrosis. Aberrant downregulation of PPAR γ is correlated with the development of fibrosis in skin, lung, pancreas, heart, and liver [245]. PPAR γ□ expression is decreased in lung fibrosis [246], liver fibrosis [247], kidney fibrosis [248] and scarring alopecia fibrosis [249]. Furthermore, reduced PPAR γ expression precede fibrosis in several human diseases, suggesting a causal role [25, 250]. Fibroblasts, with a decreased level of PPAR γ, present an increase expression of TGF-β1, type 1 collagen, and α-SMA [251, 252].

Several studies have shown, that PPAR γ agonists can diminish pro-fibrotic signal-induced collagen synthesis and can blunt fibrosis [25, 241, 245, 251, 253–257]. PPAR γ natural and synthetic ligands, such as 15d-PGJ2 and rosiglitazone, can decrease the fibroblast-myofibroblast differentiation, synthesis of collagen and firbonectin and decrease the expression of TGF-β1 [247, 248, 253, 258, 259]. Moreover, 15d-PGJ2 and rosiglitazone decrease bleomycin-induced lung fibrosis [260]. PPAR γ agonists stop the TGF-β1-induced EMT of alveolar epithelial cells and decrease fibrosis in numerous organs such as heart [261, 262], lung [263, 264], liver [265, 266] and kidney [267, 268].

#### PPAR γ and radiation-induced fibrosis

Radiation therapy causes a downregulation of PPAR γ expression [59]. PPAR γ levels are decreased in 3 to 12 hours after a total body irradiation of C57BL/6 mice [59], after UVB irradiation in skin models [269] and in 3 days following a single abdominal dose of 10 Gy [270].

Oxidative stress and inflammation are involved in radiation-induced brain injury [271, 272]. Administration of PPAR γ agonists, such as pioglitazone, can decrease the severity of radiation-induced cognitive impairment in rat models [273]. PPAR γ ligands block the radiation-induced activation of NF-κB [274]. PPAR γ agonists administration can contribute to reduce the inflammation and oxidative stress [248, 275].

PPAR γ ligands enhance radiation-induced DNA damage in lung cancer cells *in vitro* [276, 277].

PPAR γ activators can prevent irradiation-induced inflammatory processes through the inhibition of NF-κB expression and the downregulation of STAT-3 pathway, an activator of canonical WNT/β-catenin pathway [270]. Diosmin, a citrus bioflavonoid which has antioxidant, anti-inflammatory and anti-apoptotic properties [278], can increase PPAR γ expression and can decrease canonical WNT/β-catenin pathway to attenuate radiation-induced hepatic fibrosis [9].

### Interactions between PPAR γ and canonical WNT/β-catenin pathway

WNT/β-catenin pathway and PPAR γ act in an opposite manner in several diseases such as cancers [24, 279–284]. WNT/β-catenin pathway and PPAR γ interact through a TCF/LEF β-catenin domain and a catenin-binding domain within PPAR γ [285–288]. Downregulation of the WNT/β-catenin pathway induces PPAR γ stimulation while PPAR γ agonists decrease β-catenin expression in numerous cellular systems [235, 236, 289]. PPAR γ agonists can act as neuroprotective agents and promoting synaptic plasticity through a WNT/β-catenin/PI3K/Akt pathway interaction [290]. The regulation of mesenchymal stem cell differentiation also shows the existence of this crosstalk [291].

Indeed, in several diseases, β-catenin signaling inhibits PPAR γ [292–301], and using PPAR γ agonists is considered as promising treatment through this interplay [302]. Troglitazone, a PPAR γ agonist, can decrease c-Myc levels [303]. In intestinal fibrosis, the activation of WNT/β-catenin has been observed and the use of PPAR γ agonist can inhibit WNT/β-catenin pathway activation and then can repress fibrosis [304]. PPAR γ agonists stimulate Dickkopf-1 (DKK1) activity, which inhibits the canonical WNT pathway and then block fibroblast differentiation [305]. During adipogenesis in 3T3-L1 cells, PI3K/Akt pathway decreases PPAR γ expression [306]. Akt signaling participates to PPAR γ inhibition in adipocyte differentiation [307–309]. PI3K/Akt pathway phosphorylates GSK-3β and inhibits it, which decreases PPAR γ expression [176, 310]. β-catenin signaling, through its activation of Akt signaling, inhibits PPAR γ expression in adipocytes and 2T2-L1 preadipocytes [289, 311]. Moreover, inhibition of Akt pathway in 3T3-L1 cells activates the expression of PPAR γ [312]. PPAR γ agonists can decrease the activity of Akt signaling [313, 314]. Rosiglitazone and pioglitazone, PPAR γ agonists, inhibit both Akt signaling and GSK-3β activity in cardiac fibrosis to block TGF-β–induced collagen accumulation [315].

### Interactions between PPAR γ and TGF-β1

TGF-β1 upregulation decreases PPAR γ expression in fibroblasts [245] and hepatic stellate cells [316], whereas PPAR γ agonists can directly inhibit TGF-β1 activity [317], prevent expression and synthesis of collagen in fibroblasts [253, 254, 257], and then prevent α-SMA expression [253, 257]. In addition, thiazolidinediones can reduce hepatic fibrosis through the inhibition of α-SMA and TGF-β expression [318]. PPAR γ agonists can inhibit both the Smad-dependent and Smad-independent TGF-β1 pathways.

### Interactions through the Smad pathway

Anti-fibrotic PPAR γ effects can be largely explained through the Smad pathway. PPAR γ promoter has two Smad binding elements and inhibition of PPAR γ favors canonical Smad2/3 signaling [316].

In human hepatic stellate cells, PPAR γ agonists can inhibit TGF-β1/Smad3 signaling [319]. Smad-dependent collagen production is suppressed by PPAR γ ligand-activated through targeting the p300 transcriptional coactivator [254]. CDDO can prevent TGF-β1-induced fibrosis by inhibition of Smad transcription and Akt pathway [257]. TG-Interacting Factor (TGIF) which is a Smad transcriptional co-repressor can be activated by PPAR γ agonists, such as troglitazone, ciglitazone, and 15d-PGJ2. This leads to repress TGF-β1-induced fibrosis in hepatocyte cells [320, 321]. TGF-β1-induced JNK pathway is decreased by troglitazone, which decreases Smad2 signaling and then impairs myofibroblasts differentiation [322]. In addition, adiponectin prevents fibrosis in liver in mice [323, 324].

Moreover, a PPAR γ downregulation shows an increase of (increases) Smad3 and α-SMA expression and an overproduction of collagen (collagen overproduction) [252]. Rosiglitazone, a PPAR γ agonist, could decrease fibrosis by stimulating Smad7, an inhibitor of TGF/Smad signaling pathway [325]. In liver fibrosis, overexpression of prolyl oligopeptidase (POP) by S17092 (a POP inhibitor) leads to activate Smad7 protein and PPAR γ and then inactivates TGF-β signaling [326].

### Interactions through the non-Smad pathway

PPAR γ agonists can induce diminution of TGF-β1expression in alveolar epithelial cells and in tumor metastasis without interacting with the Smad pathway [327, 328]. In an independent Smad pathway manner, PPAR γ represses TGF-β1-induced fibrosis [253, 258, 317]. TGF-β1 expression can be inhibited by 15d-PGJ2 through a PTEN-mediated p70 ribosomal S6 kinase-1 inhibition [329]. PPAR γ agonists can decrease TGF-β1-induced myofibroblast differentiation via the inhibition of PI3K/Akt pathway [330]. PPAR γ agonists can stimulate PTEN expression for having anti-fibrosis effects [219, 329, 331], and leading to the inhibition of collagen production and myofibroblast differentiation [332].

### Interactions between PPAR γ and canonical WNT/β-catenin pathway/TGF-β1 in radiation-induced fibrosis (cf. Figure 3)

Several studies have shown a crosstalk between TGF-β1, canonical WNT/β-catenin pathway and PPAR γ [25, 250]. Indeed, canonical WNT/β-catenin is activated by TGF-β1, and this results in inhibition of PPAR γ. In liver fibrosis, TGF-β1 inhibits PPAR γ expression by increasing β-catenin expression [299]. In turn, activation of PPAR γ inhibits WNT/β-catenin pathway and then TGF-β1. PPAR γ targets PI3K/Akt for repress TGF-β1-induced myofibroblast differentiation [330]. PPAR γ agonists can inhibit fibrosis development through inactivation of TGF-β1 [333]. Moreover, 15-deoxy-delta12,14-prostaglandin J2, troglitazone, and rosiglitazone can suppress corneal myofibroblasts in eyes [294]. In addition, PPAR γ agonists can protect against excessive fibrosis [245]. Basic fibroblast growth factor (bFGF) can be repressed by using rosiglitazone and pioglitazone [313]. Inhibition of Akt pathway attenuates TGF-β signaling in rat kidney epithelial cells [334]. Troglitazone, a PPAR γ agonist, inhibits TGF-β signaling by phosphorylating Akt pathway at Ser437. LY294002, a PI3K inhibitor, inhibits Akt phosphorylation and α-SMA induction and then attenuates TGF-β1 signaling [335]. TGF-β-induced phosphorylation of β-catenin at Tyr654 associated with a conjunction between β-catenin and Smad3 stimulate α-SMA expression during EMT [336]. In fibrosis, troglitazone can inhibit TGF-β signaling by inhibiting β-catenin and PI3K/Akt pathway activation and by activating GSK-3β, the inhibitor of WNT pathway [335].

**Figure 3:**
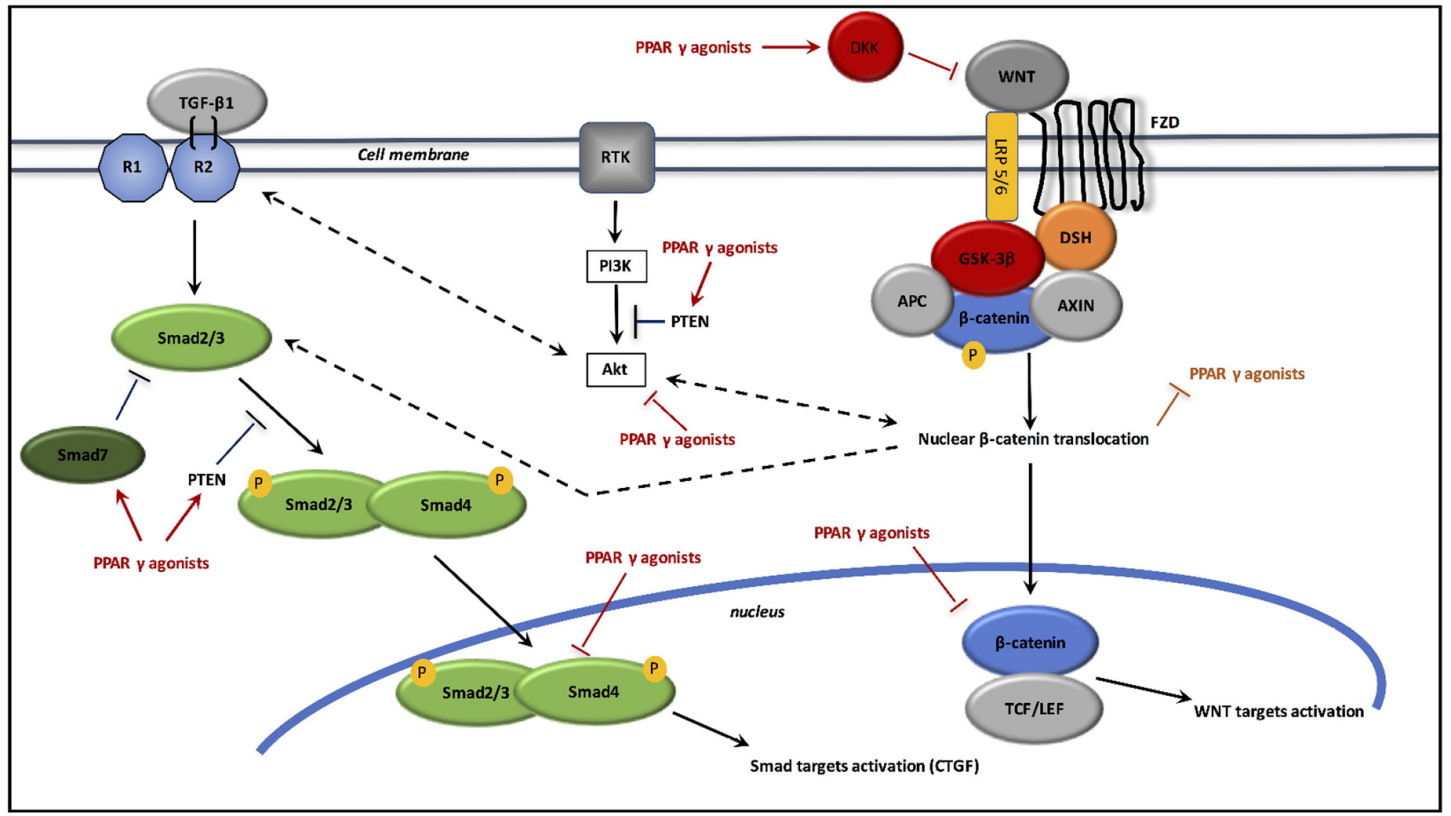
Interactions between TGF-β1, canonical WNT/β-catenin pathway and PPAR γ agonists in radiation-induced fibrosis Inflammation activates WNT ligands. WNT ligand binds FZL and LRP5/6 receptors. This leads to inactivate the destruction complex AXIN/APC/GSK-3β. β-catenin phosphorylation is thus stopped, which prevents its degradation in the proteasome. Then, β-catenin accumulates in the cytosol and translocates to the nucleus for binding TCF/LEF co-transcription factor for inducing WNT target genes such as c-Myc and cyclin D1. WNT pathway and PI3K/Akt pathway increase each other. Inflammation and DNA damage also activate TGF-β1, which induces the Smad pathway. TGF-β1 binds type 2 TGF-β receptor, which recruits type 1 TGF-β receptor. The heterotetramer formed phosphorylates Smad2/3, which binds to Smad4. The Smad complex translocates to the nucleus for activation its target genes, such as CTGF. PPAR γ agonists activate Dickkopf-1 (DKK) for the inhibition of WNT ligands, and prevent β-catenin accumulation by activating GSK-3β. PPAR γ agonists inhibit Akt activity and stimulate PTEN, the inhibitor of PI3K. PPAR γ agonists also stimulate Smad7 and PTEN for inhibiting Smad pathway. Adenomatous polyposis coli (APC); dishevelled (DSH); frizzled (FZD); glycogen synthase kinase-3β (GSK-3β); low-density lipoprotein receptor-related protein 5/6 (LRP5/6); peroxisome proliferator-activated receptor γ (PPARγ); TGF-β1 receptors 1 and 2 (R1, R2); T-cell factor /lymphoid enhancer factor (TCF/LEF); transforming growth factor (TGF); phosphatase and tensin homolog (PTEN).

## CONCLUSION

**Figure 4:**
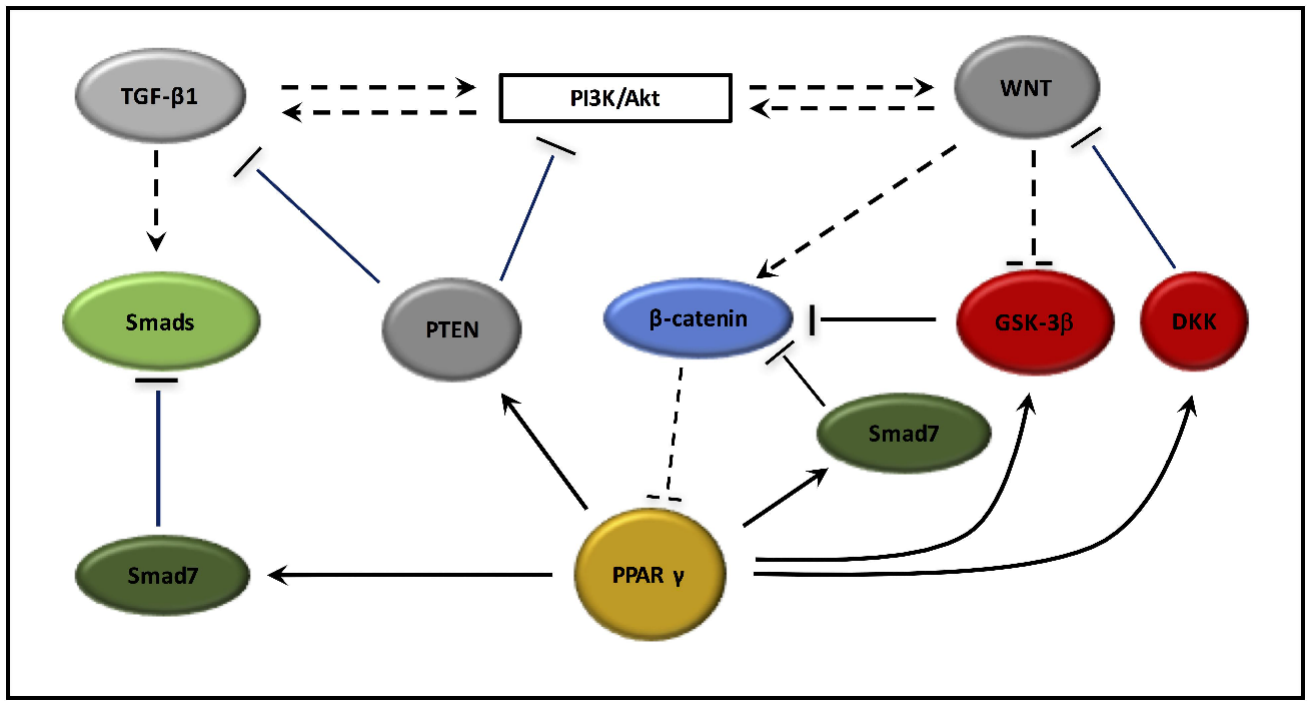
PPAR γ interactions with TGF-β1, PI3K/Akt pathway and the canonical WNT/β-catenin pathway PPAR γ agonists activate Smad7, an inhibitor of Smads pathway. PPAR γ agonists activate PTEN, which inhibits both PI3K/Akt pathway and TGF-β1 signaling. PPAR γ agonists activate DKK and Smad7, two inhibitors of WNT/beta-catenin pathway leading to the β-catenin degradation in the proteasome. PPAR γ agonists activate GSK-3β activity, which forms the destruction complex to degrade β-catenin. In contrast, presence of WNT ligands inhibits GSK-3β and lead to cytosolic accumulation of β-catenin. Then nuclear β-catenin associates with TCF/LEF in the nucleus to inhibit PPAR γ expression.

Radiation-induced fibrosis is characterized by DNA damage and inflammation. These two processes lead to the activation of TGF-β1 and canonical WNT/β-catenin pathway. TGF-β1 plays a major role in the differentiation of fibroblasts into myofibroblasts. Myofibroblasts appear able to physiological contraction and relaxation. However, in fibrosis myofibroblasts generate a phenomenon of contraction-retraction lasting without relaxation and with an irreversible retraction favored by the synthesis of collagen. Myofibroblasts show a main role in cellular fibrosis in numerous organs such as kidney, heart, lung, liver and wound. TGF-β1 operates in a canonical WNT/β-catenin pathway dependent manner. These two pathways stimulate each other through the Smad pathway or non-Smad pathways like PI3K/Akt pathway. TGF-β1 stimulates myofibroblast differentiation by the stimulation of canonical WNT pathway and the downregulation of PPAR γ expression. TGF-β1 appears to be an interesting therapeutic target in fibrosis [337, 338]. PPAR γ agonists stimulate Smad7 for inhibiting Smads pathway what blocks TGF-β signaling. PPAR γ agonist can decrease canonical WNT/β-catenin pathway by activating both Smad 7, GSK-3β and DKK. PPAR γ agonists can also stimulate PTEN, an inhibitor of PI3K/Akt pathway and inhibit the Smad pathway for downregulation TGF-β1 expression. Thus, by both inhibiting TGF-β1 and the canonical WNT/β-catenin pathway, PPAR γ agonists could be interesting preventive targets for radiation-induced fibrosis treatment (cf. Figure 4).

## Abbreviations

APC: adenomatous polyposis coli
DSH: disheveled
FZD: frizzled
GSK-3β: glycogen synthase kinase-3β
LRP 5/6: low-density lipoprotein receptor-related protein 5/6
PI3K-Akt: phosphatidylinositol 3-kinase-protein kinase B
PPAR γ: peroxisome proliferator-activated receptor γ
RIF: radiation-induced fibrosis
TCF/LEF: T-cell factor/lymphoid enhancer factor
TGF-β: transforming growth factor β
